# Exploration of an Efficient Electroporation System for Heterologous Gene Expression in the Genome of Methanotroph

**DOI:** 10.3389/fmicb.2021.717033

**Published:** 2021-08-04

**Authors:** Lizhen Hu, Shuqi Guo, Xin Yan, Tianqing Zhang, Jing Xiang, Qiang Fei

**Affiliations:** ^1^School of Chemical Engineering and Technology, Xi’an Jiaotong University, Xi’an, China; ^2^Key Laboratory of Agricultural Environmental Microbiology, Ministry of Agriculture, College of Life Sciences, Nanjing Agricultural University, Nanjing, China; ^3^Shaanxi Key Laboratory of Energy Chemical Process Intensification, Xi’an Jiaotong University, Xi’an, China

**Keywords:** one-carbon substrate, site-specific chromosome expression, transformation efficiency, gene deletion, heterologous gene expression, methanotroph

## Abstract

One-carbon (C1) substrates such as methane and methanol have been considered as the next-generation carbon source in industrial biotechnology with the characteristics of low cost, availability, and bioconvertibility. Recently, methanotrophic bacteria naturally capable of converting C1 substrates have drawn attractive attention for their promising applications in C1-based biomanufacturing for the production of chemicals or fuels. Although genetic tools have been explored for metabolically engineered methanotroph construction, there is still a lack of efficient methods for heterologous gene expression in methanotrophs. Here, a rapid and efficient electroporation method with a high transformation efficiency was developed for a robust methanotroph of *Methylomicrobium buryatense* 5GB1. Based on the homologous recombination and high transformation efficiency, gene deletion and heterologous gene expression can be simultaneously achieved by direct electroporation of PCR-generated linear DNA fragments. In this study, the influence of several key parameters (competent cell preparation, electroporation condition, recovery time, and antibiotic concentration) on the transformation efficiency was investigated for optimum conditions. The maximum electroporation efficiency of 719 ± 22.5 CFU/μg DNA was reached, which presents a 10-fold improvement. By employing this method, an engineered *M. buryatense* 5GB1 was constructed to biosynthesize isobutyraldehyde by replacing an endogenous *fadE* gene in the genome with a heterologous *kivd* gene. This study provides a potential and efficient strategy and method to facilitate the cell factory construction of methanotrophs.

## Introduction

Methane, derived from natural gas and biogas, is the second most abundant greenhouse gas whose global warming potential is 25 times more than that of carbon dioxide (Fei et al., 2014; Pariatamby et al., 2015). Excessive methane emissions can not only cause a waste of carbon sources but also endanger the environment by causing the global warming effect (Bjorck et al., 2018; Hu et al., 2020). Thus, it is urgent to seek a potential, green, and sustainable strategy for the efficient utilization of methane.

With the development of molecular biology, the bioconversion of methane into chemicals or biofuels by special industrial microbial catalysts has become a promising trend for its mitigation (Nguyen and Lee, 2020). Compared with chemical methods, the biological routes of methane utilization are relatively simple with the potential to directly activate methane at ambient temperature and atmospheric pressure (Conrado and Gonzalez, 2014). Methanotrophs are capable of utilizing methane as the sole energy and carbon source, which also constituted the main biocatalysts for the production of C1-based chemicals or biofuels (Hur et al., 2017; Su et al., 2017; Nguyen et al., 2018). Notably, the methanotroph *Methylomicrobium buryatense* 5GBl, which can utilize one-carbon (C1) substrates (methane and methanol) for growth, has been studied extensively and in depth due to its promising characteristics for industrial application, including a fast growth rate, strong anti-contamination ability, a robust endogenous methane assimilation pathway, and the availability of genetic manipulation tools and bioreactor (Alexey et al., 2015; Yan et al., 2016; Fei et al., 2018). Currently, conjugation-based transformation plays an important role in achieving gene transfer and expression in *M. buryatense* 5GBl (Puri et al., 2015), which usually requires a helper strain such as *Escherichia coli* S17-1 λpir to transfer DNA into a host due to the restriction–modification (R-M) system (Yan et al., 2016). Nevertheless, the time-consuming elimination of *E. coli* after conjugation limited the use of this method. Hence, to broaden the industrial application of *M. buryatense* 5GBl in C1 substrate conversion, many gaps still need to be further solved, especially the development of efficient genetic manipulation methods for the metabolic engineering of methanotrophs.

Electroporation, as a direct gene transfer system, is widely used in various bacteria for DNA transfer (Itoh et al., 1994). It is reported that the key steps such as the preparation of competent cells, electroporation process, recovery process, and plating screening mainly affect the number of transformants and the transformation efficiency during the electroporation process (Fu et al., 2017; Morales-Ruiz et al., 2019). Moreover, compared with conjugation-based transformation methods, the electroporation method is relatively easy to perform for directly and efficiently inserting DNA fragments into specific sites of the genome to complete the heterologous gene expression. Because the R-M system of *M. buryatense* 5GBl can be interfered by the plasmid transfer during electroporation, it could be an ideal choice to introduce linear DNA fragments by electroporation for efficiently achieving gene deletion and plasmid-free gene expression. In addition, the marker system Flp/FRT and a markerless system using the counterselectable marker *sacB* or *pheS* have been used for chromosomal modification in methanotrophs (Liu et al., 2021), but an available and efficient method to establishing a common site for completing heterologous gene expression in the genome of *M. buryatense* 5GBl is still required.

This study aimed to develop a site-specific chromosome expression (SSCE) method with an efficient electroporation system for methanotrophs using an antibiotic-selected marker due to its stability (Yan et al., 2016). Since the deletion of beta-oxidation did not show a negative influence on the growth of *M. buryatense* 5GB1 (Demidenko et al., 2016), the position of the *fadE* gene on the chromosome was selected as a specific site. The key parameters of the gene manipulation method, such as the DNA concentration, cell density, methanol concentration, recovery time, field strength, and the antibiotic concentration, that affect the transformation efficiency of *M. buryatense* 5GBl were investigated and optimized in this study. To demonstrate the feasibility of this novel electroporation-based method, the gene deletion and heterologous gene expression strategies in *M. buryatense* 5GBl were established by artificially replacing the endogenous *fadE* gene with the heterologous *kivd* gene for isobutyraldehyde biosynthesis. Finally, the performances of the plasmid-based method and the SSCE method were analyzed and compared in order to evaluate the specific site of *fadE* for exogenous gene expression.

## Materials and Methods

### Strains, Plasmid, Culture, and Antibiotic Screening

The strains and plasmids used in the study are listed in Table 1. Competent cells (*E. coli* DH5α) were cultured on Luria broth (LB) agar plates and *M. buryatense* 5GBlS were stocked on nitrate mineral salt (NMS) agar medium with 1% methanol. Liquid cultures of 50 ml were used for culturing *M. buryatense* 5GBlS in 250-ml flasks in a shaker at 30°C and 200 rpm. The mating plate (NMS2) consisted of 85% NMS and 15% LB agar culture media. The concentrations of the carbonate buffer and the phosphate buffer were individually adjusted to 5 and 5.8 mM, respectively, for the conjugation process, according to a previous report (Puri et al., 2015). Methanol, which could fulfill the requirements of genetic manipulation for the growth of *M. buryatense* 5GBlS, was utilized as the carbon source in all methanotrophic cultures instead of methane to simplify the procedure of cultivation (Hu and Lidstrom, 2014; Wang et al., 2020). The antibiotic concentrations used for screening colonies were as follows: kanamycin (Km^r^), 50–100 μg/ml; gentamicin (Gm^r^), 10–40 μg/ml. All experiments were carried out in duplicate or triplicate. Data were processed and analyzed with SPSS 18.0 software, and *P*-values with statistical significance at *P* < 0.05 were obtained.

**TABLE 1:** List of the strains and plasmids used in the study.

Strains and plasmids	Description	References
*E. coli* DH5α	*E. coli*, F^–^, φ80, *lac*ZΔM15, Δ(*lac*ZYA-*arg*F) U169 *end*A1, *rec*A1, *hsd*R17 (r_k_^–^, m_k_^+^) supE44, λ^–^, *thi*-1, *gyr*A96, *rel*A1, *pho*A	Sangon Biotech Co. (Shanghai, China)
*E. coli* DH5α-*kivd*	Variant of *E. coli* DH5α, Km^r^, pAWP89 vector containing *kivd*	This study
*E. coli* DH5α (pRK600)	Variant of *E. coli* DH5α, Cm^r^, triparental conjugation helper	This study
*M. buryatense* 5GBlS	Variant of *M. buryatense* 5GB1, capable of being conjugated with small IncP-based plasmid	Puri et al., 2015
*M. buryatense* 5GBlSΔ*fadE*	*M. buryatense* 5GBlSΔ*fadE*:Km^r^ (MBURv2_190114)	This study
*M. buryatense* 5GBlSΔ*fadE*:Km^r^:*kivd*	Variant of *M. buryatense* 5GB1SΔ*fadE*; Km^r^ and *kivd* were attached to the *fadE* site in the genome.	This study
*M. buryatense* 5GBlSΔ*fadE*:Gm^r^:*pos*5	Variant of *M. buryatense* 5GB1SΔ*fadE*; Gm^r^ and *pos*5 were attached to the *fadE* site in the genome.	This study
*M. buryatense* 5GBlS-pAWP89:*kivd*	Variant of *M. buryatense* 5GB1S, containing pHLZ66	This study
pAWP89	IncP-based broad host range plasmid containing dTomato, Km^r^	Puri et al., 2015
pHLZ66	Variant of pAWP89 containing *kivd*, Km^r^	This study

### DNA Manipulation

The plasmid pAWP89 was separated from *E*. *coli* DH5α with an AxyPrep Plasmid Miniprep Kit (Axygen, Suzhou, China) and linearized by NSPl (NEB, Beijing, China). The concentration of DNA was measured by the DS-11 spectrophotometer (DeNovix, Wilmington, DE, United States). The oligonucleotides used to amplify the DNA fragments are listed in Supplementary Table 1. The complex (DNA fragment) was obtained by overlap PCR. The fragments were retrieved using MonPure Gel and PCR Clean Kit (Monad Biotech, Wuhan, China). The transformants were identified by two oligonucleotides of the target gene, and a 2 × Rapid Taq Master Mix (Vazyme, Nanjing, China) was used. The Ezup Column Bacteria Genomic DNA Purification Kit (Sangon Biotech Co., Shanghai, China) was used to extract genomic DNA.

### Electroporation Protocol

*Methylomicrobium buryatense* 5GBlS was firstly cultured at 30°C with shaking until the optical density at 600-nm wavelength (OD_600_) reaches 2.0, checked with UV spectrophotometry (TU-1810, PERSEE, Beijing, China). Then, the cells in the logarithmic phase were collected and 50 ml of the bacterial solution was centrifuged to remove supernatants (5,000 × *g*, 10 min at 4°C), then cleaned three times with ice-cold water (autoclave sterilization, 121°C for 20 min). Finally, methanotroph competent cells were completely dissolved with 500 μl deionized water, and 50 μl of competent cells was mixed with 100, 200, 400, 600, 800, or 1,000 ng of DNA fragment. For the electroporation process, 1-mm-gap cuvettes were placed into the Gemini SC2 instrument (BTX, Holliston, MA, United States) to finish the electroporation process. After electroporation, 1 ml of fresh NMS was added to the cuvette, which was transferred into the 10 ml NMS medium in 250-ml serum bottles. Different concentrations of methanol (0.02–0.5%) were added to the resuscitation medium to culture the cells. The cells were collected and transferred into plates containing different concentrations of kanamycin after 3, 6, 9, 12, and 24 h of resuscitation. The plates were incubated for 4 days at 30°C for screening of transformants. The cell density (1.0, 2.9, 4.9, 9.7, and 19.4 × 10^11^ CFU/ml), field strength (12, 15, 18, and 25 kV/cm), and recovery time (3, 6, 9, 12, and 24 h) were also explored individually in the study for higher transformation efficiency by electroporation.

The basic conditions used in this method were as follows: cell density, OD_600_ = 0.4–0.6; DNA concentration, 500–1,000 ng; field strength, 12–20 kV/cm; methanol concentration, 0.02–0.5% in NMS medium; and resistance concentration, 50–100 μg/ml (Yan et al., 2016; Nguyen et al., 2018; Liu et al., 2021). According to the conditions mentioned above, the basic transformation parameters set as the standard conditions in this experiment were as follows: cell density, 4.9 × 10^11^ CFU/ml; DNA concentration, 600 ng; electric field strength, 15 kV/cm; methanol concentration, 0.1%; and resistance concentration, 50 μg/ml. Among them, a cell density of 4.9 × 10^11^ CFU/ml was obtained according to the initial cell density given in the reference (OD_600_ = 0.4; 50 ml culture solution was concentrated to 200 μl) (Nguyen et al., 2018).

### Method for Recombinant Plasmid Transfer

*Escherichia coli* DH5α, *M. buryatense* 5GBlS, and *E. coli* DH5α (PRK600) were involved in the conjugation process. NMS2 mating plates were prepared according to a previous literature (Puri et al., 2015). Firstly, we transferred the recombinant plasmid pHLZ66 into *E. coli* DH5α (donor bacteria). Secondly, one loop of *M. buryatense* 5GBlS was evenly coated in NMS2 mating plates (15% LB + 85% NMS) and cultured overnight at 30°C. Then, *E. coli* DH5α and PRK600 were cultured on LB medium overnight at 37°C. Equal volumes of *E. coli* DH5α and *E. coli* DH5α (PRK600) were evenly spread on NMS2 mating plates and cultured at 30°C for 2 days. One loop of mixed cells was transferred to a selective NMS plate with kanamycin (100 μg/ml) for the selection of transconjugants, which were then transferred into a new selective NMS plate.

### Isobutyraldehyde Detection

The concentration of isobutyraldehyde was determined using Agilent Technologies 7890A GC System (Agilent Technologies, Santa Clara, CA, United States) with a DB-Wax column (30 m × 0.32 mm × 0.5 μm; Agilent Technologies) and a flame ionization detector. The flow rates of hydrogen, air, and nitrogen were 30, 400, and 25 ml/min, respectively. The oven temperature was held at 35°C for 5 min, then heated to 230°C at a rate of 12°C/min, and ending at 230°C. The retention time of isobutyraldehyde was 1.667 min. A standard curve was obtained after commercial isobutyraldehyde was treated with the sample with the above method.

## Results

### Establishment of a Fast Electroporation Method for Endogenous Gene Deletion

**FIGURE 1:**
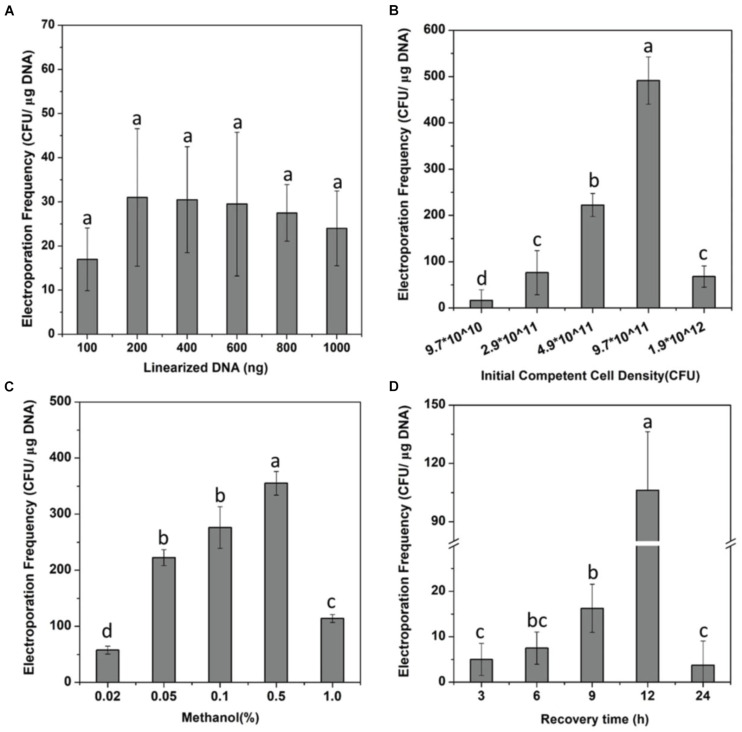
Optimization of the electroporation system. **(A)** Effect of the number of linearized DNA fragments on the electroporation efficiency of strain *Methylomicrobium buryatense* 5GBlSΔ*fadE*. **(B–D)** Effects of the cell density **(B)**, methanol concentration **(C)**, and the recovery time **(D)** on the electroporation efficiency of strain *Methylomicrobium buryatense* 5GB1S. *Lowercase letters above bars* indicate significant differences (*P* < 0.05).

Although it has been shown that DNA transfer can be achieved through electroporation into methanotrophs (Yan et al., 2016; Nguyen et al., 2018; Liu et al., 2021), optimum conditions of the electroporation system for *M. buryatense* 5GBlS are still needed to ensure a higher transformation efficiency of gene deletion before developing the SSCE method. After obtaining the *fadE* gene deletion complex (PCR product) for the construction of *M. buryatense* 5GBlSΔ*fadE* by electroporation (Supplementary Figure 1), the influence of six different DNA concentrations on the number of transformants was firstly investigated. As can be seen in Figure 1A, the number of transformants was less than 20 at a DNA concentration of 100 ng, which increased to up to 30 at 200 ng, which is in good agreement with a previous report (Yekta et al., 2013). However, the number of transformants showed a plateau effect after using 400 ng DNA, which was finally selected for subsequent experiments.

The cell density can also affect the transformation efficiency, showing a positive relation in Figure 1B. At a cell density of 3.9 × 10^11^ CFU/ml, the transformation efficiency increased to 14 times higher than that of 1.0 × 10^11^ CFU/ml; the highest transformation efficiency in *M. buryatense* 5GBlSΔ*fadE* of 491 CFU/μg DNA was achieved at a cell density of 9.7 × 10^11^ CFU/ml, which can be considered as a statistically significant effect. These findings can be explained by the higher cell concentration indirectly promoting the efficiency of transformation by increasing the probability of binding with DNA. Nevertheless, when the cell density was 1.9 × 10^12^ CFU/ml, the transformation efficiency was less than 100 CFU/μg DNA. Methanol, as an essential carbon source, was explored for cell recovery and growth after electroporation. As shown in Figure 1C, when the concentration of methanol added into NMS increased from 0.02 to 0.5%, the transformation efficiency was enhanced from less than 60 to up to 400 CFU/μg DNA. These results revealed that a higher methanol concentration is needed to improve the transformation efficiency by promoting the growth and reproduction of transformants. But it is worth noting that only a few transformants were observed with the concentration of 1%, which may be due to the inhibition effect of methanol.

In addition, studies have shown that the field strength and antibiotic concentration also affected the efficiency of transformation because a high field strength during electroporation is likely to destabilize the cell walls of Gram-positive bacteria and different antibiotic concentrations can affect the growth and reproduction of transformants (Dower et al., 1988; Mcintyre and Harlander, 1989; Steele et al., 1994; Shimogawara et al., 1998; Min et al., 2018). However, our data showed that field strength of 12–25 kV/cm and antibiotic concentrations of 50–100 μg/ml did not provide significant improvements in the transformation efficiency of *M. buryatense* 5GBlS (data not shown). The process of recovery is a stage of cell growth, reproduction, damage repair, and resistance gene expression (Li et al., 2014). Therefore, a relatively long recovery benefits the rapid growth of cells and the successful recombination of exogenous DNA fragments into the genome. However, a satisfactory transformation efficiency can only be achieved with a recovery time of 12 h (Figure 1D). In the end, the electroporation conditions used for the SSCE method were as follows: cell density, OD_600_ = 9.7 × 10^11^ CFU/ml; field strength, 18 kV/cm; recovery time, 12 h; carbon source concentration, 0.5%; and kanamycin concentration, 100 μg/ml. Finally, the highest transformation efficiency for *fadE* gene deletion was 719 ± 22.5 CFU/μg DNA, which was 10 times more than that of the initial condition (66.25 CFU/μg DNA).

### Development of the SSCE Method for Heterologous Gene Expression

**FIGURE 2:**
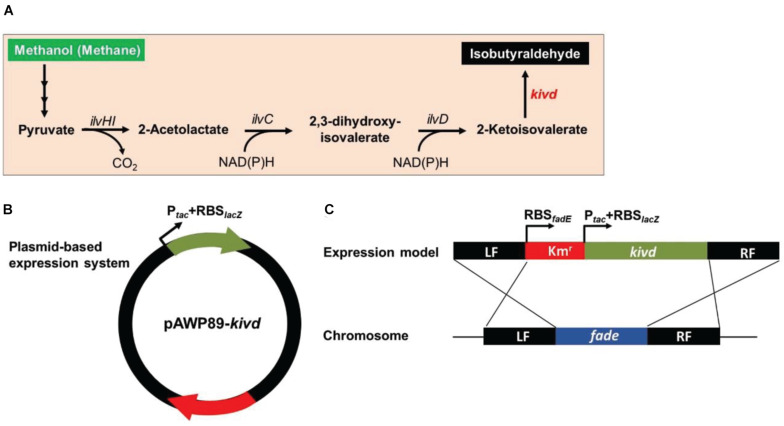
Framework of the heterologous expression of 2-ketoisovalerate decarboxylase (KivD) in strain *Methylomicrobium buryatense* 5GB1S. **(A)** Artificial pathway for isobutyraldehyde production from methanol or methane in strain *M. buryatense* 5GB1S. **(B)** Scheme of the plasmid-based expression of the foreign gene *kivd* in strain *M. buryatense* 5GB1S. **(C)** Scheme of the integration of the foreign gene *kivd* into the chromosome of strain *M. buryatense* 5GB1S. *Word in red* represents an exogenous gene. *Words in black* represent endogenous genes. These genes coded for acetohydroxyacid synthase (*ilvHI*), acetohydroxyacid isomeroreductase (*ilvC*), and dihydroxyacid dehydratase (*ilvD*). *Intersecting lines* indicate homologous recombination. *LF*, left flanking region; *RF*, right flanking region.

**FIGURE 3:**
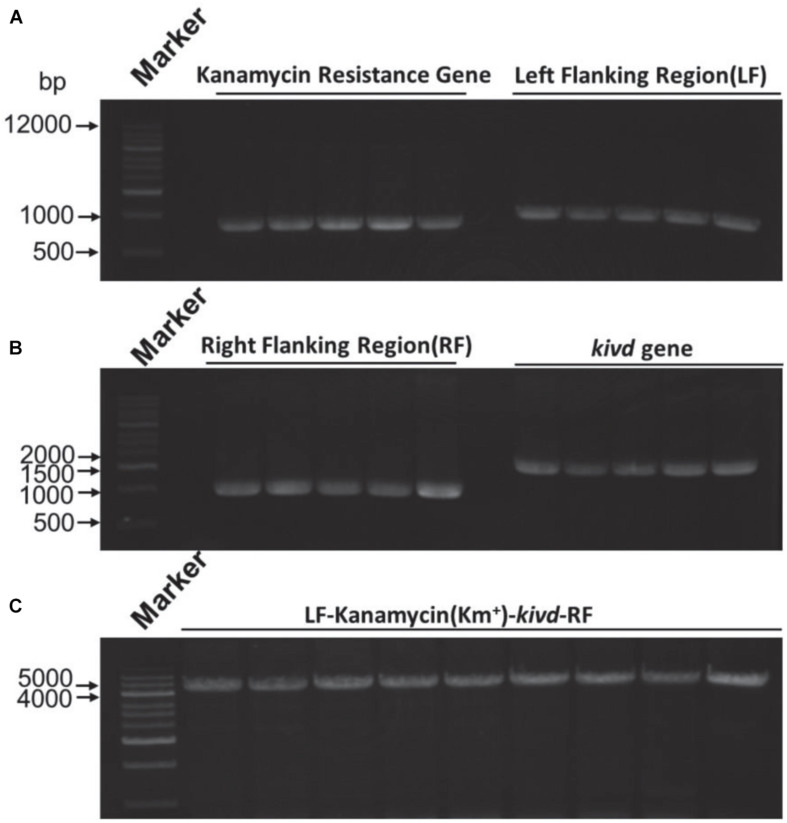
Expression of the *kivd* gene in the *fadE* site of strain *Methylomicrobium buryatense* 5GB1S. **(A)** Agarose gel electrophoresis results of the kanamycin gene (816 bp) and the left flanking region (1,000 bp) for *kivd* expression. **(B)** Agarose gel electrophoresis results of the right flanking region (1,000 bp) for *kivd* expression of the kanamycin gene (1,000 bp) and the *kivd* gene (1,719 bp). **(C)** PCR confirmation of the expression complex of the *kivd* gene (LF + Km^r^ + *kivd* + RF). A 1-kb marker was used. *LF*, left flanking region; *RF*, right flanking region; *Km*^r^, kanamycin.

To evaluate the SSCE method, *M. buryatense* 5GBlS was engineered for the biosynthesis of isobutyraldehyde, which is the key precursor of isobutanol applied as an attractive fuel substitute (Atsumi et al., 2010). The gene encoding alpha-ketoisovalerate decarboxylase (KivD) from *Lactococcus lactis*, which can catalyze 3-methyl-2-oxybutyric acid to isobutyraldehyde, was used as the heterologous gene, as shown in Figure 2A. Two strategies can be employed for the expression of the heterologous gene *kivd* in methanotrophs: a plasmid-based expression (Figure 2B) and a chromosome-based homologous recombination (Figure 2C). Although the former has been applied in many model strains with better outcomes, the R-M system in methanotrophs results in a lower efficiency. Therefore, a modified chromosome-based method was developed to provide a fast and efficient system. As shown in Figure 3 and Supplementary Figure 2, the recombinant strain (*M. buryatense* 5GBlSΔ*fadE*:Km^r^:*kivd*) was constructed by replacing the specific site of the *fadE* gene in the genome with the exogenous DNA fragment of *kivd* and Km^r^. Furthermore, to verify the validity of the *fadE* gene site as an effective and specific site for heterologous gene expression, the *pos*5 gene, for regulating the reducing power level and promoting product accumulation from *Saccharomyces cerevisiae* coding for NADH kinase along with the Gm^r^ fragment, was also introduced into the *fadE* site, giving the recombinant strain of *M. buryatense* 5GBlSΔ*fadE*:Gm^r^:*pos*5 successfully (Supplementary Figures 3–5).

**FIGURE 4:**
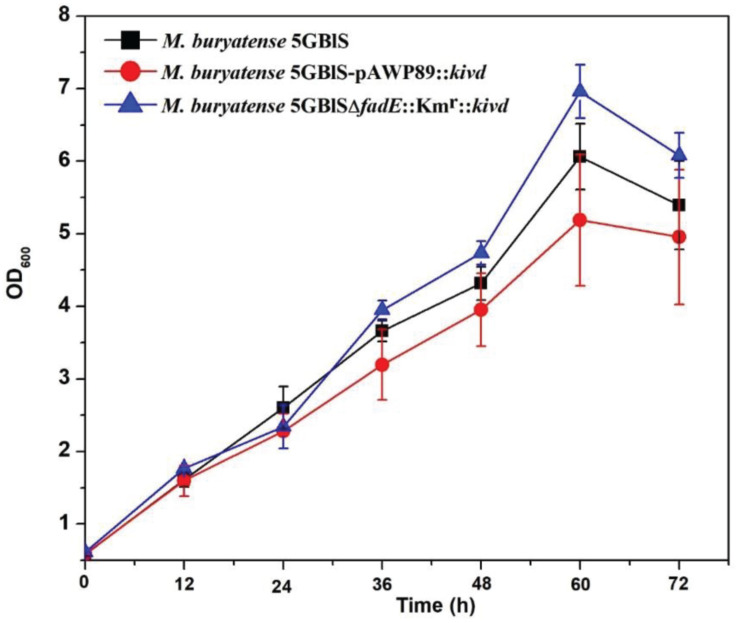
Growth performance of the wild-type (*Methylomicrobium buryatense* 5GB1S) and mutant strains for isobutyraldehyde biosynthesis.

To investigate the expression efficiency of the exogenous gene insertion at the *fadE* site, a plasmid-based recombinant (*M. buryatense* 5GBlS-pAWP89:*kivd*) was constructed for comparison. As shown in Figure 4, *M. buryatense* 5GBlSΔ*fadE*:Km^r^:*kivd*, with the best situation for growth, displayed the highest OD_600_ of 7.0, followed by OD_600_ = 6.0 for *M. buryatense* 5GBlS (wild type) and OD_600_ = 5.0 for *M. buryatense* 5GBlS-pAWP89:*kivd*. The biosynthesis of isobutyraldehyde was also validated in the recombinants and wild type. As shown in Table 2, it is clear that the wild-type strain could not accumulate isobutyraldehyde due to the lack of the *kivd* gene. Because of the successful overexpression of the heterologous *kivd* gene, the accumulation of isobutyraldehyde was obtained in both recombinants with a similar titer around 3.3 mg/L. Overall, the SSCE method with the *fadE* site was developed as a promising and plasmid-free strategy for heterologous gene expression with chromosome integration.

**TABLE 2:** Biosynthesis of isobutyraldehyde in the cultures of wild-type (*Methylomicrobium buryatense* 5GB1S) and mutant strains.

Strains	Titer (mg/L)			
	**0 h**	**24 h**	**48 h**	**72 h**
*M. buryatense* 5GBlS	0.00	0.00	0.00	0.00
*M. buryatense* 5GBlS-pAWP89:*kivd*	0.00	3.08 ± 0.18	3.53 ± 0.04	3.01 ± 0.24
*M. buryatense* 5GBlSΔ*fadE*:Km^r^:*kivd*	0.00	2.94 ± 0.10	3.29 ± 0.07	2.89 ± 0.20

## Discussion

To obtain powerful cell factories, faster and more convenient genetic tools are essential. Electroporation-based transformation is a simple and efficient method for the genetic engineering modification of microorganisms, so improving the DNA transfer efficiency of electroporation is conducive to the rapid and efficient construction of engineered bacteria, which can be applied in industrial applications of methanotrophs. In *M. buryatense* 5GB1S, C1 substrates are taken up and bioconverted into ketoisovalerate, which is the main precursor for the synthesis of isobutyraldehyde (Matsuda et al., 2013; Miao et al., 2018).

To complete the heterologous gene expression, an efficient electroporation system based on *M. buryatense* 5GB1S was firstly optimized. Key factors including the cell density, methanol concentration, and recovery time were investigated based on previous reports (Puri et al., 2015; Yan et al., 2016; Nguyen et al., 2018; Liu et al., 2021). Therein, the cell density also showed a positive relation with the electroporation efficiency of *M. buryatense* 5GB1S, and this is consistent with previous reports (Holo and Nes, 1989; Shimogawara et al., 1998; Wu and Letchworth, 2004). For example, researchers have explored the influence of the DNA concentration on the transformation efficiency of fibroblasts and found that the transformation efficiency was increased by 17 times with the DNA concentration increasing fourfold (Yekta et al., 2013). Moreover, it was reported that the ability of electroporation to transfer DNA into cells was limited under a fixed cell concentration, and excess DNA remained inefficient (Hattermann and Stacey, 1990; Rittich and Španová, 1996). It is believed that a high substrate concentration will facilitate cell growth and improve efficiency. It has been reported that a high methanol concentration in the electroporation system could easily trigger cell lysis, resulting in low conversion efficiency (Yan et al., 2016). Consequently, similar findings were observed when the methanol concentration was 1.0%, providing a dramatically low efficiency. Furthermore, an appropriate recovery time is beneficial to the improvement of the transformation efficiency, which could severely affect the conversion efficiency due to blocking of the efficient entry of linear DNA into the cell and difficulty in the screening of transformants (Hattermann and Stacey, 1990; Yan et al., 2016). The longer recovery time in this study also showed lower efficiency mainly due to cell lysis (Yan et al., 2016).

The optimized SSCE method developed in this research showed unique advantages in gene deletion and heterologous gene expression in methanotrophs compared with the existing methods. A higher transformation frequency was observed when using PCR-generated fragments to delete the *glgA2* (0.37 kb) and *mmo* (0.83 kb) genes in methanotrophs through electroporation (Yan et al., 2016), which may be due to the shorter length of the selected genes compared with that of *fadE* (2.3 kb). Thus, the frequency of 719.0 ± 22.5 CFU/μg DNA obtained from this *fadE*-based SSCE was still high enough for most genetic manipulations, including gene deletion and heterologous gene expression in *M. buryatense* 5GBlS. Moreover, the antibiotic cassette used in the *fadE*-based SSCE method was less than 1 kb in comparison to the markerless counterselection cassettes *pheS* and *sacB* used in methanotrophs, which can facilitate the overlap step during PCR (Nguyen and Lee, 2020). Finally, the biosynthesis of isobutyraldehyde was conducted by heterologously expressing *kivd* in *M. buryatense* 5GB1S with deletion of the *fadE* gene. Favorably, better growth was achieved with a similar isobutyraldehyde titer from the SSCE method compared with the plasmid-based method because of the metabolic burden of the plasmid. Nevertheless, the yield of isobutyraldehyde on cell density from *M. buryatense* 5GBlSΔ*fadE*:Km^r^:*kivd* (0.69 mg/L per OD_600_) was lower than that of *M. buryatense* 5GBlS-pAWP89:*kivd* (0.89 mg/L per OD_600_), which may be due to the higher copy number from a plasmid than a single copy of the inserted gene. Thus, optimization of the promoters or the ribosome-binding site (RBS) for this *fadE*-based SSCE method is required in future works in order to deliver a robust strategy for the genetic engineering of methanotrophs.

In this work, an efficient and rapid method was developed to delete the endogenous gene and complete the heterologous gene expression in *M. buryatense* 5GB1S simultaneously. The exogenous gene *kivd* was inserted into the genome of *M. buryatense* 5GBlS (*fadE*), forming an isobutyraldehyde producer. Moreover, the *fadE* site could be used as an effective and specific site for the exogenous expression, with better growth due to the plasmid-free system. Overall, this study not only provides a bright idea for gene deletion and expression but also enriches the scope of genetic tools for methanotrophs, which promotes the application of methanotrophs in the utilization of C1 substrates.

## Data Availability Statement

The original contributions presented in the study are included in the article/Supplementary Material, further inquiries can be directed to the corresponding author/s.

## Author Contributions

QF and LH conceived and designed the work and provided conceptual advice with inputs from all authors. LH planned and performed the experiments and analyzed the data. LH, SG, and QF wrote and revised the manuscript. TZ and JX helped to complete the work. SG and XY provided conceptual advice. All authors contributed to the data analyses and read, revised, and approved the final manuscript.

## Conflict of Interest

The authors declare that the research was conducted in the absence of any commercial or financial relationships that could be construed as a potential conflict of interest.

## Publisher’s Note

All claims expressed in this article are solely those of the authors and do not necessarily represent those of their affiliated organizations, or those of the publisher, the editors and the reviewers. Any product that may be evaluated in this article, or claim that may be made by its manufacturer, is not guaranteed or endorsed by the publisher.
