# Supplementary material for: Exploration of an Efficient Electroporation System for Heterologous Gene Expression in the Genome of Methanotroph
Source: Front Microbiol. 2021 Aug 4;12:717033. doi: 10.3389/fmicb.2021.717033 (PMC8373458; doi:10.3389/fmicb.2021.717033)

**Supplementary material**

**Exploration of an efficient electroporation system for heterologous gene expression in the genome of methanotroph**

Lizhen Hu^1†^, Shuqi Guo^1†^, Xin Yan^2^,Tianqing Zhang^1^, Jing Xiang^1^, Qiang Fei^1*^

^1^School of Chemical Engineering and Technology, Xi'an Jiaotong University, Xi'an, China;

^2^ Key Laboratory of Agricultural Environmental Microbiology, Ministry of Agriculture, College of Life Sciences, Nanjing Agricultural University, Nanjing, China

*Corresponding author:

Qiang Fei: feiqiang@xjtu.edu.cn

^†^Lizhen Hu and Shuqi Guo contributed equally to this work.

Funding

The National Key R&D Programs of China (2018YFA0901500) and National Natural Science Foundation of China (21878241), and the Key Research and Development Program of Shaanxi Province (2021SF-103).

**The supplementary materials include:**

**Supplementary tables and figures**

**Table S1** List of primers and sequences for amplification used in the study.

**Figure S1** The *fadE* gene deleting. (A) Scheme for gene deletion strategy in strain *M. buryatense* 5GB1S. (B) PCR construction of *fadE* gene deleting fragment.

**Figure S2** Verification of transformants by PCR..

**Figure S3** The agarose gel electrophoresis results. Lane 1-3, 751 bp, Gm^r^; lane 4-6, 1317 bp, pos5; Lane 7-9, 1000 bp, LF; Lane 10-12,1000 bp, RF; Lane 13, 1 kb marker.

**Figure S4** The agarose gel electrophoresis results. Lane 1-10, 4068 bp, LF+Gm^r^+*pos*5+RF; Lane 11, 1 kb Marker.

**Figure S5** Verification of transformants by PCR.

**Table S1** List of primers and sequences for amplification used in the study.

| **Primers** | **DNA** | **Sequence (5'→3')** |
| --- | --- | --- |
| LF1-F | LF1 | CGGCATTGCTGCGGCAA |
| LF1-R | LF1 | GTTGAATATGGCTCATGGCTTAATTCCTGTTG |
| K1-F | K1^+^ | CAACAGGAATTAAGCCATGAGCCATATTCAAC |
| K1-R | K1^+^ | TTGCTAATCAGGTGAAGTTAGAAAAACTCATCGAGCA |
| RF1-F | RF1 | TGCTCGATGAGTTTTTCTAACTTCACCTGATTAGCAA |
| RF1-R | RF1 | AAATTGTCCGACATCGACCTGGC |
| LF2-F | LF2 | CGGCATTGCTGCGGCAAGATTGGG |
| LF2-R | LF2 | CGTTTCCCGTTGAATATGGCTCATGGCTTAATTCCTGTTGCTGTG |
| K2-F | K2^+^ | CACAGCAACAGGAATTAAGCCATGAGCCATATTCAACGGGAAACG |
| K2-R | K2^+^ | ATTGTCAACAGCTCATTTCAGAGTTAGAAAAACTCATCGAGCAT |
| kivd2-F | kivd 2 | ATGCTCGATGAGTTTTTCTAACTCTGAAATGAGCTGTTGACAAT |
| kivd 2-R | kivd 2 | TGAAGCATCTTGCTAATCAGGTGAAGTCACGATTTATTTTGTTCCGCA |
| RF2-F | RF2 | TGCGGAACAAAATAAATCGTGACTTCACCTGATTAGCAAGATGCTTCA |
| RF2-R | RF2 | AAATTGTCCGACATCGACCTGGC |
| Kivd3-F | Kivd3 | tattcacacaggaaacagctATGTATACCGTTGGCGATTATTTG |
| Kivd3-R | Kivd3 | acgcatcttcccgacaactaTCACGATTTATTTTGTTCCGCA |
| P89-F | pAWP89 | TAGTTGTCGGGAAGATGCGT |
| P89-R | pAWP89 | AGCTGTTTCCTGTGTGAATA |
| LF3-F | LF3 | CGGCATTGCTGCGGCAAGATTGG |
| LF3-R | LF3 | GAACCGAACAGGCTTATGTCAAGGCTTAATTCCTGTTGCTGTGT |
| G-F | Gm^r^ | ACACAGCAACAGGAATTAAGCCTTGACATAAGCCTGTTCGGTTC |
| G-R | Gm^r^ | GTCAACAGCTCATTTCAGAGTTAGGTGGCGGTACTTGGGT |
| POS5-F | Pos5 | ACCCAAGTACCGCCACCTAACTCTGAAATGAGCTGTTGAC |
| POS5-R | Pos5 | ATCTTGCTAATCAGGTGAAGTCAATCATTATCGGTTTGTC |
| RF3-F | RF3 | GACAAACCGATAATGATTGACTTCACCTGATTAGCAAGAT |
| RF3-R | RF3 | AAATTGTCCGACATCGACCTG |

LF1, K1, and RF1 for knock out model.

LF2, K2, kivd2, and RF2 for expression model (*kivd*).

LF2, K2, kivd2, and RF2 for expression model (*pos*5).

Kivd3 and p89 for expression by plasmid.

**Figure S1** The *fadE* gene deleting. (A) Scheme for gene deletion strategy in strain *M. buryatense* 5GB1S. (B) PCR construction of *fadE* gene deleting fragment. **
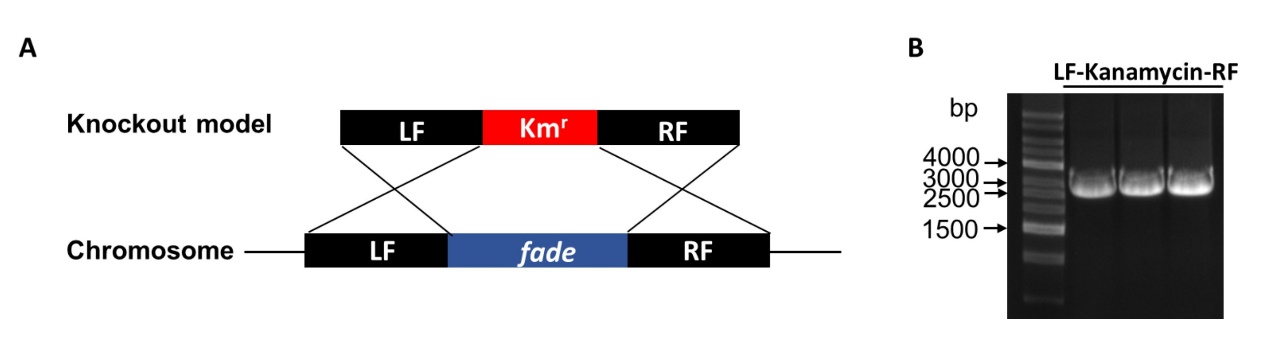
**

**Figure S2** Verification of transformants by PCR.


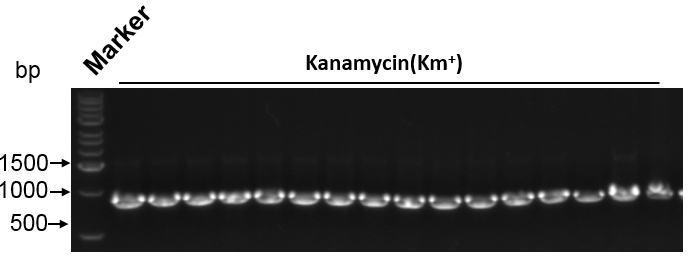


26 27 28 29 30 31 32 33 34 35 36 37 38 39 40 41 42 43 44 45 46 47 48 49 50

**Figure S3** The agarose gel electrophoresis results. Lane 1-3, 751 bp, Gm^r^; Lane 4-6, 1317 bp, *pos*5; Lane 7-9,1000 bp, LF; Lane 10-12,1000 bp, RF; Lane 13, 1 Kb marker.


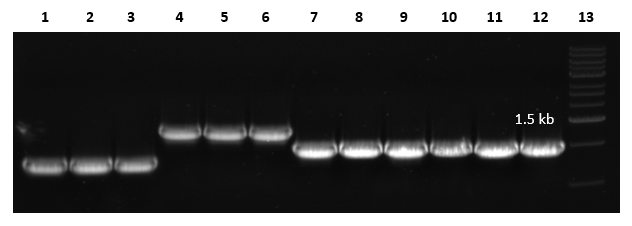


**Figure S4** The agarose gel electrophoresis results. Lane 1-10, 4068 bp, LF+Gm^r^+*pos*5+RF; Lane 11, 1 Kb Marker.


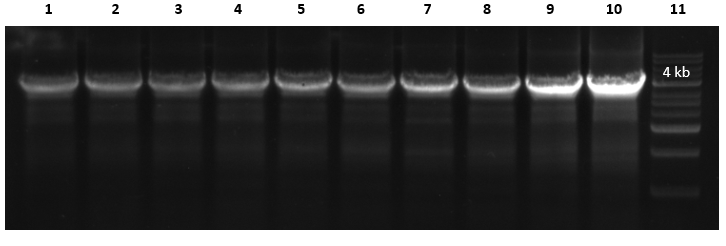


**Figure S5** Verification of transformants by PCR.


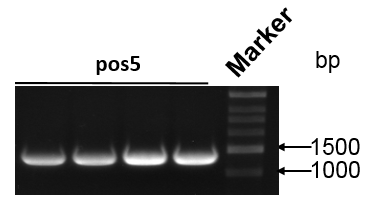

Supplement: Supplementary file 1 [file Data_Sheet_1.docx]
